# Sperm lacking Bindin are infertile but are otherwise indistinguishable from wildtype sperm

**DOI:** 10.1038/s41598-021-00570-6

**Published:** 2021-11-03

**Authors:** Gary M. Wessel, Yuuko Wada, Mamiko Yajima, Masato Kiyomoto

**Affiliations:** 1 Division of BioMed, Department of Molecular and Cellular Biology and Biochemistry, Brown University, 185 Meeting Street, Providence, RI 02912 USA; 2 Tateyama Marine Laboratory, Marine and Coastal Research Center, Ochanomizu University, Kou-yatsu 11, Tateyama, Chiba 294-0301 Japan

**Keywords:** Cell biology, Developmental biology, Evolution, Zoology

## Abstract

Cell–cell fusion is limited to only a few cell types in the body of most organisms and sperm and eggs are paradigmatic in this process. The specialized cellular mechanism of fertilization includes the timely exposure of gamete–specific interaction proteins by the sperm as it approaches the egg. Bindin in sea urchin sperm is one such gamete interaction protein and it enables species–specific interaction with a homotypic egg. We recently showed that Bindin is essential for fertilization by use of Cas9 targeted gene inactivation in the sea urchin, *Hemicentrotus pulcherrimus*. Here we show phenotypic details of Bindin-minus sperm. Sperm lacking Bindin do not bind to nor fertilize eggs at even high concentrations, yet they otherwise have wildtype morphology and function. These features include head shape, tail length and beating frequency, an acrosomal vesicle, a nuclear fossa, and they undergo an acrosomal reaction. The only phenotypic differences between wildtype and Bindin-minus sperm identified is that Bindin-minus sperm have a slightly shorter head, likely as a result of an acrosome lacking Bindin. These data, and the observation that Bindin-minus embryos develop normally and metamorphose into normal functioning adults, support the contention that Bindin functions are limited to species–specific sperm–egg interactions. We conclude that the evolutionary divergence of Bindin is not constrained by any other biological roles.

## Introduction

Sperm are highly specialized cells. Most of their organelles have been jettisoned during development, including a majority of their cytoplasm, mitochondria, and their highly condensed nuclei even have only one copy of each chromosome. In most organisms studied, sperm also have a long flagellar tail that functions with characteristic beat dynamics, and a specialized secretory vesicle, the acrosomal vesicle, that is stimulated for secretion by extracellular factors of the egg. The contents of the acrosomal vesicle, upon their release, empowers the sperm to penetrate the egg extracellular matrix, and to reach the egg cell surface for binding and fusion. Each of these specializations in a sperm enhance interaction with an egg^1–3^.

Disruption of genes expressed in sperm often cause catastrophic changes in the process of spermatogenesis, complicating the conclusion of direct gene function. A knock out of the mouse spermatocyte-specific heat shock protein HSP70-2, for example, causes meiotic failure, germ cell apoptosis, and infertility in male mice^4^. Disruption of the RNA-binding protein Tenr, which is expressed solely in germ cells of the testis in mice, have a reduced sperm count, decreased motility, malformed heads, and inability to penetrate the zona pellucida^5^. Knock out of *Gopc* (Golgi-associated PDZ- and coiled-coil motif-containing protein) results in poorly formed acrosomes, globozoospermia, defects in the mitochondrial sheath, and infertility in a mouse^6^.

On the other hand, inactivation of some genes leads to normal appearing sperm with a singular deficit. For example, inactivation of the Catsper gene causes deficiencies in sperm motility, but are otherwise morphologically similar to their wildtype sibling sperm^7^. Catsper is a calcium channel located specifically in the sperm tail. Upon ligand binding, calcium influx through the Catsper channel induces hyperactivation of the sperm, enabling sperm to identify the location and reach the egg.

Izumo is an Ig-domain containing transmembrane protein in the mammalian sperm acrosome and, following the acrosome reaction, is responsible for sperm binding to the egg cell surface^8^. Loss of Izumo causes no apparent defect in the general health of the adult males, and shows no difference in the movement, morphology, or mechanism in the sperm’s ability to reach the egg. But, these Izumo-null sperm are infertile.

The first species–specific gamete interaction protein identified was Bindin of the sea urchin. Bindin was discovered in the sperm acrosome, and is released upon sperm activation^2^. Intense study of this protein has resulted in many hypotheses as to its role in sperm function, for interaction, and perhaps for fusion with the egg. Divergence of Bindin sequence is also thought to be a driving force in speciation^8–13^.

Bindin is a single copy gene in the sea urchins whose genomes have been sequenced. It is translated as a pre-pro-protein, considered to be targeted into the endoplasmic reticulum, and then packaged into the acrosomal secretory vesicle, coincident with cleavage of the pro-protein region. Upon maturation it is an insoluble protein and its isolation results in particles of Bindin, facilitating its use for egg binding assays in vitro using agglutination as a measure of its egg-binding activity^1^.

Recently, we reported Bindin functionality in vivo in the sea urchin *H. pulcherrimus* by use of Cas9-gRNA gene targeting and learned that the resultant Bindin-null sperm were incapable of fertilizing eggs^3^. Here we characterize the phenotypes of Bindin-null sperm and conclude that the sole function of Bindin is in sperm–egg interactions. This focused, yet essential function, therefore may enable rapid divergence in this gene to function in gene drift for fertilization, and in genetic segregation, resulting in reproductive isolation.

## Results

**Figure 1:**
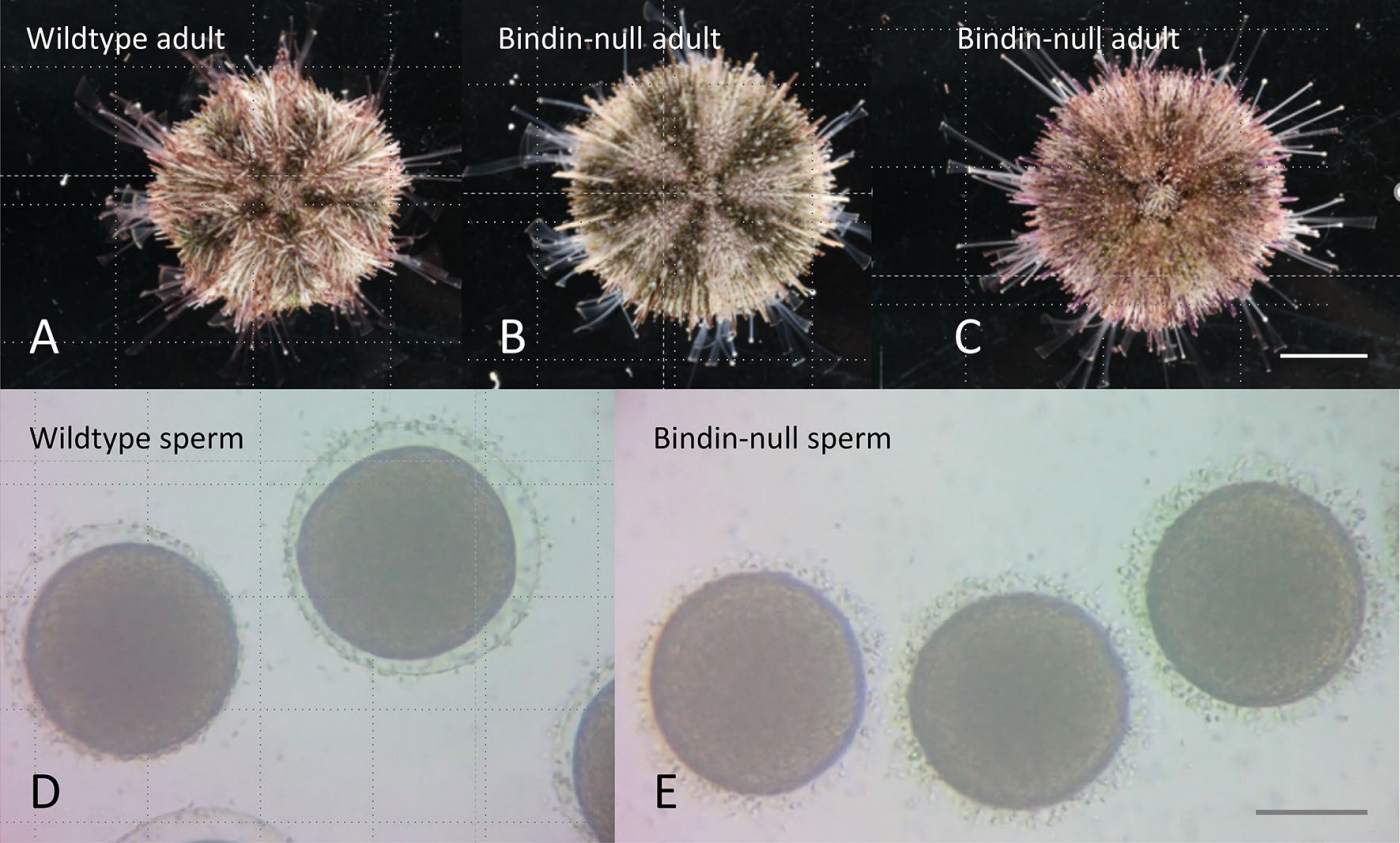
A wildtype adult H. pulcherrimus seen from the dorsal (aboral) surface (**A**). Bindin-null adults (**B** and **C**) are indistinguishable from the wildtype adults, and throughout their developmental progression to this point (not shown). Wildtype sperm activate eggs within moments of exposure, resulting in formation of the fertilization envelop (**D**). In contrast, Bindin-null sperm never activate an egg, even after prolonged exposure (**E**). See also Video S1 in the Supplemental Material. Scale bar in **C** = 1 cm for **A**–**C**; Scale bar in **E** = 40 µm for **D** and **E**.

**Figure 2:**
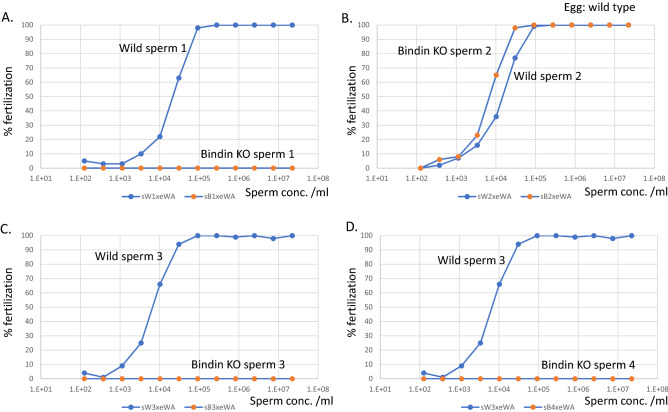
Fertilization assays using increasing sperm concentrations (from 100 sperm to over 10 million sperm per milliliter). Eggs of a single female were used for sperm from each male (**A**–**D**) throughout these assays. Two independent cycles of experiments were performed and one cycle of the dataset is shown. Bindin #2 represents sperm from an adult whose embryos were injected with Cas9 and gRNAs, yet no mutations were detectable in this animal. (Male B2 in Table #1).

Four gRNAs were designed to target the pro-region of the predicted *bindin* gene in the sea urchin *H. pulcherrimus* (Hp; Figs. S1–S5; Hpbase: cell-innovation.nig.ac.jp/Hpul/). These gRNAs along with the mRNA encoding Cas9 were injected into freshly fertilized eggs of Hp, and cultured as for sibling wildtype or Cas9-only controls. Development was indistinguishable between these cultures to adulthood, and only upon a fertilization test, was a distinction noticed; bindin-null sperm did not bind to nor fertilize eggs (Fig. 1; Video S1; Wessel et al., 2021). At a variety of sperm concentrations, up to 50 million sperm per milliliter, eggs were never fertilized in multiple and distinct mutational genotypes (Fig. 2; see also Table 1). Even heterospecific *H. crassispina* sperm activated Hp eggs better than Bindin-null Hp sperm (Fig. S6). A summary of the bindin mutations tested is shown in Tables 1 and 2.

**Table 1:** Tabulation of mutations and phenotypes.

Individual	Genotype	Phenotype
Male B1	Mutations at gRNA 373 and gRNA 455	Infertile *No Bindin
Male B2	No mutations	Fertile *Normal Bindin
Male B3	Mutations at gRNA 373 and gRNA 455	Infertile *No Bindin
Male B4	Mutations at gRNA 373	Infertile *No Bindin
Male B5	Mixed mutation in gRNA 455, 2 base deletion in gRNA 373, both yield inactive product	Infertile
Male B6	9 base deletion in gRNA 82, not a change in ORF	Fertile
Male B7	Mutation at site gRNA 325, 5 base deletion, frame shift	Infertile
Male B8	6 base deletion and 2 amino acid changes in gRNA 373, does not affect the ORF	Infertile

**Table 2:** Tabulation of mutations and phenotypes.

gRNA	Male individuals mutated	Female individuals mutated
82	B6 mutation but does not change ORF	B1, 2, and have a frameshift mutation
325	B7	None
373	B1, B3, B4 (although B1 mutation does not shift ORF), B5, B8	B3 has a frameshift mutation
455	B1, B3, B5, B7	None

Overall: 10/11 animals microinjected with Cas9/gRNAs had mutations (91%), 2 of the mutations did not disrupt the open reading frame (20%), and 4 of the 10 had mutations at multiple gRNA sites (40%). The useful mutation rate was thus 73%.

*No Bindin refers to results of immunoblots and immunolocalization data in Wessel et al., 2021).

**Figure 3:**
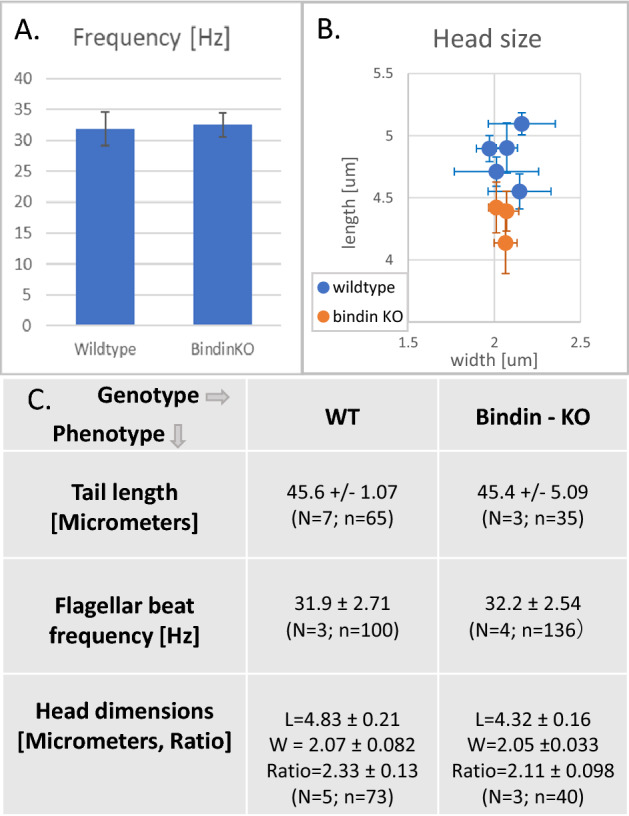
Phenotypic comparison of Wildtype and *bindin*—KO sperm. Flagellar beat frequency and tail length are nearly identical between the wildtype and Bindin-null sperm. (**A**) Average values of tail beat frequency. (**B**) Head dimensions of individual animal values. N shows number of batches measured and n shows number of sperm measured. The *bindin*-KO sperm batches used for this measurement were B1, B3, and B4 of Table 1. (**C**) Summary of quantitative sperm morphometry. Statistical significance: Tail length *p* > 0.05, flagellar beat *p* > 0.05, head length *p* < 0.005, head width *p* > 0.05, ratio of head length to head width *p* < 0.005.

**Figure 4:**
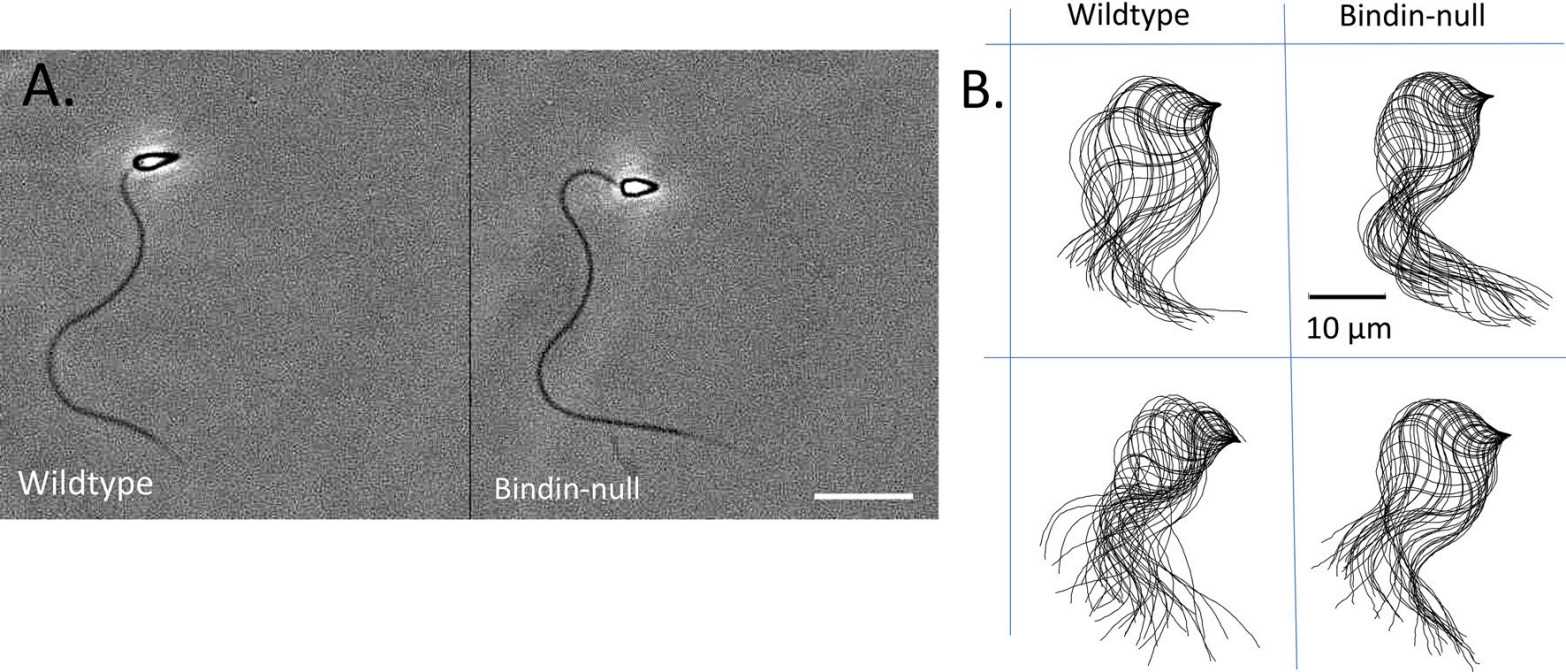
(**A**) Swimming sperm of wildtype (left) and Bindin-null (right) sperm from *Hemicentrotus pulcherrimus*. Image contrast has been enhanced using Image-J (ver.1.52a). Note that sperm from the Bindin-null sperm has similar head and tail morphometrics. (**B**) Flagellar profile from 45 successive traces of sperm. Note subtle variations between samples though essential profiles are consistent. Scale bars: 10 µm.

**Figure 5:**
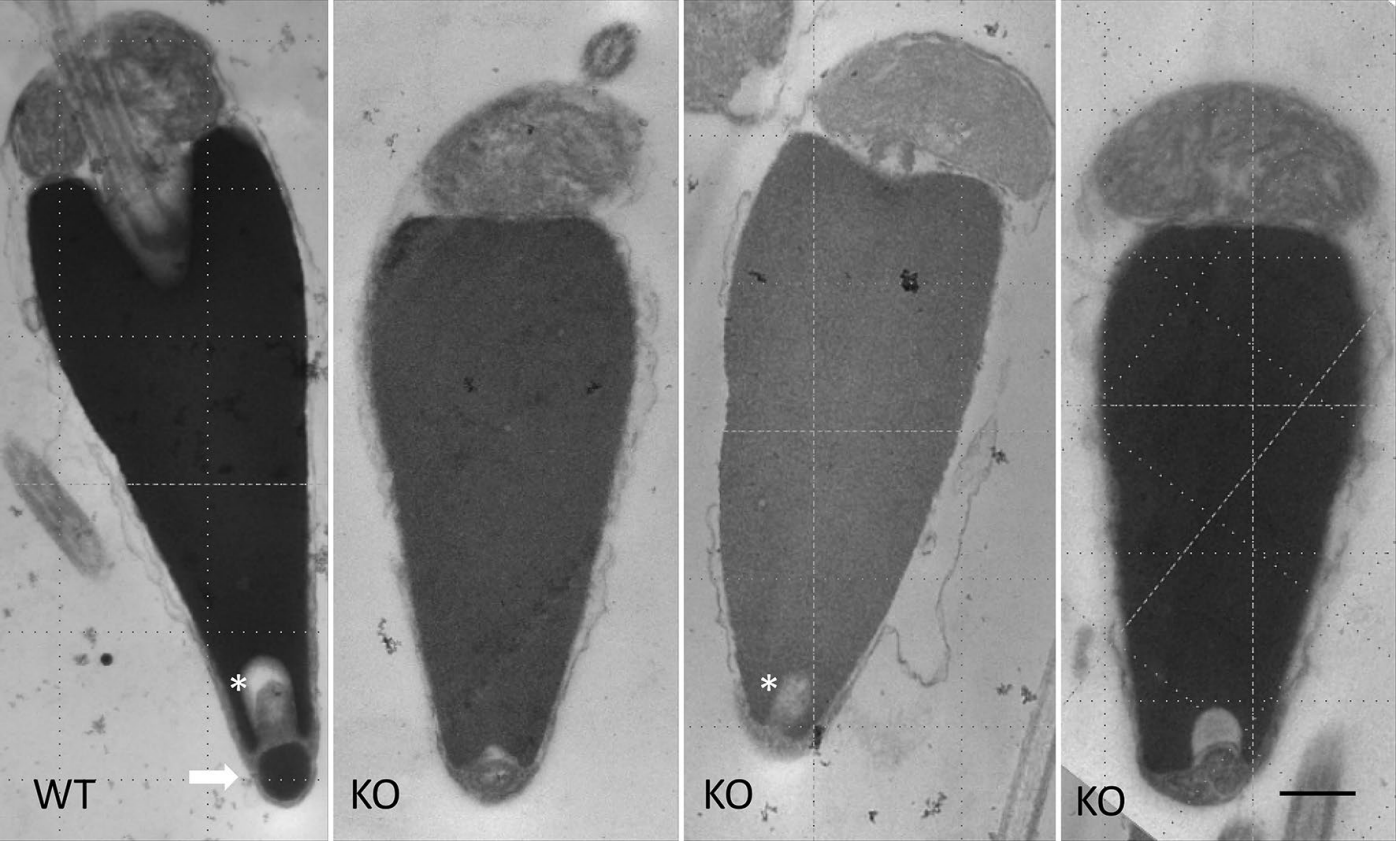
TEM of Wildtype and Bindin-null sperm. Note the acrosome of the wildtype sperm (white arrow) with densely packed Bindin. Three representative sperm from the bindin-KO adult (Bindin #3) still have an acrosomal vesicle, but it appears less dense, and vacuolated. Note the horseshoe shaped fossa (asterisks) within the sperm nucleus even in the sperm lacking bindin. Scale bar = 0.5 µm.

**Figure 6:**
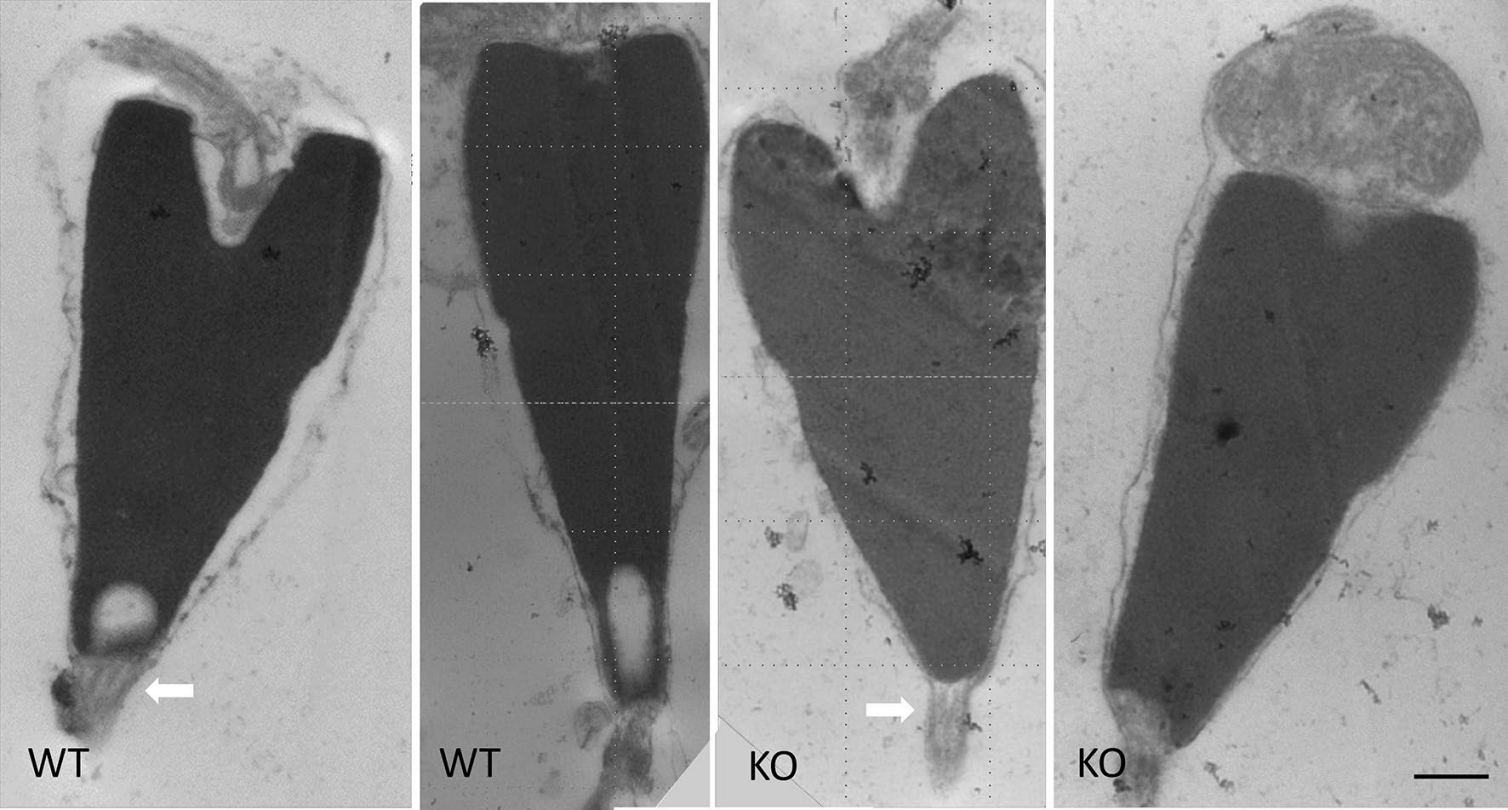
TEM of Wildtype and bindin-KO sperm following exposure to egg jelly that stimulates the acrosome reaction. Note the acrosomal process (white arrow) in both wildtype and bindin-KO. Scale bar = 0.5 µm.

Although infertile, the Bindin KO sperm otherwise looked normal by light microscopy. These Bindin-null sperm had a normal looking head, midpiece, tail, and they swam with wildtype speed and trajectory. To test more effectively the consequence of Bindin depletion, we quantitated the morphometry of the head, the tail length, and swimming dynamics (Figs. 3, 4; Video S2). We found that the head was slightly, but consistently, shorter than the head of the wildtype sperm (Fig. 3B, 4), perhaps reflecting the absence of a major protein of the acrosome located at the tip of sperm head. We noted by scanning electron microscopy (SEM) that the sperm head length was ~ 8% shorter in the *bindin* KO sperm relative to wildtype sperm (Fig. 5), complementary to the light microscope analysis (Fig. 3B). We also applied motility analysis in detail, including comparisons of tail curvature profiles and head trajectories, and found that all sperm swimming metrics were within variation of each other (Fig. 4; Figs. S8 and S9). TEM also revealed that the *bindin* KO sperm contained a normal complement of mitochondria, a centriolar fossa, normal nuclear shape, and a nuclear fossa containing what appeared to be membranous elements (Fig. 5). Both TEM and scanning electron microscopy (SEM) also suggest that the Bindin-null sperm underwent the acrosome reaction upon exposure to egg jelly, the same as seen in wildtype sperm (Fig. 6; Fig. S7). All other features measured in the Bindin-null sperm were indistinguishable from a wildtype sperm, so our conclusion is that infertility in these sperm is attributable solely to the lack of Bindin.

## Discussion

Although limited to a small set of cell types, cell–cell fusion does occur in somatic cells of the body in many organisms. The molecular mechanisms of these fusion events, however, appear to be distinct from those of gametes. For gamete interaction, Izumo in mammalian sperm, for example, is expressed uniquely in sperm and is essential for sperm–egg binding^8^, much as hypothesized for Bindin^2,14^. As seen in the results presented here, Bindin also appears specifically and exclusively involved in egg binding; all other aspects of development, adulthood, sperm production and function in Bindin-null sperm, are indistinguishable from wildtype sperm. Even though a small amount of bindin mRNAs is present in larval development based on transcriptomics data (echinobase.org), this mRNA does not appear to be essential for development at any time.

The anterior and posterior fossa in sperm of this animal are distinct morphologically by TEM (Figs. 5, 6). The centriole occupies the posterior fossa, and through its microtubule organization function could be essential in the formation of this fossa. The centriole, its fossa, and the sperm tail of wildtype sperm are each indistinguishable in the Bindin-null sperm. The anterior fossa though, was anticipated to be in part a result of the acrosomal contents^15^. Although the Bindin-null sperm still have membranous components in the anterior, acrosomal fossa, they appear empty. Thus, we conclude that formation of the anterior fossa is not caused by a presence of substantial and constraining acrosomal contents. This structure rather appears independently of the major acrosomal protein, Bindin. We currently do not know if other elements of the acrosome, such as the acrosomal protease^16^, are still present, nor if the lamins that are selectively present in the anterior and posterior foss a^17^ remain in this region, but both may function as a signal for the fossae formation.

Importantly, each of the sperm specific egg-binding proteins identified have rapid sequence divergence, suggesting they are undergoing significant positive selection during evolution^13^. Evidence for positive selection in Izumo genes is seen in three groups of mammals studied^18–20^ and is even seen in the Izumo ortholog of turtles^21^. Rapid positive selection is also seen in Bindin of sea urchins, and its molecular divergence has been paradigmatic for studies of sympatry and speciation^9,11,12,14^.

Having a sole function in egg binding may be advantageous for rapid divergence for egg binding proteins in sperm. This logic can be supported from a counter example: genes whose proteins interact with many other proteins tend to evolve slower than those with few(er) interactions. So genes with one, focused function, even one with an essential impact on fitness, may be less constrained. The results presented here support the concept that Bindin has only a focused, non-networked function that may enable rapid molecular evolution. In the event that changes in the egg’s receptor for sperm occur as a result of sexual conflict and other evolutionary pressures, a compensatory change in sperm could occur in a function-focused gene such as Bindin, rapidly accommodating evolutionary changes without disrupting sperm-egg binding events.

## Materials and methods

### Animal culture

Eggs and sperm were collected, fertilized and cultured from spawning wild-type adults of *H. pulcherrimus* by injection of 2 mM acetylcholine into the coelomic cavity of the adult. The resultant embryos were cultured at 15 °C and the larvae were fed the diatom *Chaetoceros gracilis*, ad libitum. Metamorphosis was induced by adding pieces of plastic plate covered with calcareous red algae^22^. The resulting juveniles were cultured at 15 °C and fed dried seaweed *Undaria pinnatifida *ad libitum. Animals achieved sexual maturation in approximately 1.5 years.

### Cas9 mRNA/Guide RNAs (gRNAs) preparation and microinjection

Guide RNAs (gRNAs) were designed using CRISPRscan (www.crisprscan.org) to coding sequences of the pro-protein domain of the Hp *bindin* gene at HpBase (http://cell-innovation.nig.ac.jp/Hpul/;^23^) and synthesized as reported^24^. The plasmid pCS2-3xFLAG-NLS-SpCas9-NLS was a gift from Yonglong Chen (Addgene plasmid #51307), and was linearized with NotI and transcribed with SP6. This transcript encodes Cas9 (codon optimized for mammalian cells) along with two nuclear localization sequences (NLS;^25^) and has been shown previously to be functional in sea urchin embryos^26^. The gRNAs were synthesized by T7 RNA polymerase using the MegaShortScript T7 transcription kit (AM1354, ThermoFisher, Waltham, MA, USA) as described in CRISPRscan (www.crisprscan.org). The gRNAs were then purified using the miRNeasy Mini kit (217004, Qiagen, Valencia, CA, USA). The four gRNAs (200 ng/ul of each gRNA; shown in Fig. 1) were mixed with 500 ng/μl of Cas9 mRNA, injected into freshly fertilized eggs as described previously in^27^.

### Identification of genomic mutations

Genomic DNA was isolated from several tube feet donated by each subject using 100 µl of QuickExtract DNA Extraction Solution (http://www.epibio.com/) according to manufacturer’s instructions. One microliter of the extraction mix was then subjected to PCR amplification of the targeted genomic DNA region: 95 °C, 3 min, 95 °C, 15 s, 60 °C, 15 s, 72 °C, 30 s, 95 °C, 15 s, repeated 30 rounds. Sequence of the PCR population was accomplished using the same amplification primers and mutation sites were identified by either direct sequence or by decomposition of trace chromatograms (https://tide.deskgen.com/;^28^) or by individual clones of the gDNA amplicons.

### Measurement of the sperm beat frequency

Sperm were collected directly from the gonopores of spawning males (dry sperm) and was diluted 100× with ST-SW (Millipore filtered sea water containing 200 µg/ml sulfamethoxazole, 10 µg/ml trimethoprim, pH 7.5). The sperm suspension was further diluted an additional 100× with F-NSW (filtered natural sea water) containing 0.01% (w/v) Bovine Serum Albumin (Wako, Japan) and placed between a glass slide and a coverslip with two strips of plastic tape (Scotch, 3 M) as spacers. The frequency of flagellar beating was determined by a method described^29^, using a dark-field microscope (Olympus CH; A40 objective lens) equipped with stroboscopic illumination (modified from the original design of^30^).

### Measurement of flagellar length and head dimensions from live sperm

Images of motile spermatozoa were observed under a phase contrast microscope (UPlan Fl objective lens, IX2-LWUCD condenser on Olympus IX-51 inverted microscope) and recorded at 90 fps with a camera (UI-3130CP-M-GL, iDS, Germany) driven by control software (Boh Navi, Bohboh software, Japan) on a LAVIE HZ550/D (NEC, 64 bit, Windows 10). Flagellar length and head size from motile sperm were measured with flagellar analyzing software Bohboh (Bohboh Rel. 4.78; BohbohSoft, Tokyo, Japan;^31^). For flagellar length, 10 successive video frames were analyzed for each sperm to reduce measurement error caused by video noise and end piece orientation. First, the flagellar profile was digitized by the auto-tracking tool in Bohboh and flagella length was calculated using these data. The flagellar length of 7–10 sperm were measured for each sea urchin batch. These flagellar profile data from successive 45 frames were used for beat form comparison as well. For head size, 5 successive frames from two different orientations (total 10 images each) were used for each sperm to reduce measurement error due to head orientation. Width and length of each sperm head was measured by fitting a rectangle to the head image using the draw tool of Bohboh and each rectangular profile was collected. From 10 to 20 data points were collected from each sperm batch.

### Ultrastructural analysis of sperm

Sperm and eggs were fixed either in 5% paraformaldehyde/2% glutaraldehyde in 80% filtered seawater, or in 2% osmium tetroxide in 50% filtered seawater for 20 min. Samples for SEM were layered on a 12 mm glass cover slip that had been coated with a 1% solution of protamine sulfate (Sigma, P-4020) for 10 min and then washed with deionized water. After the fixed samples had settled on the coverslip (15 min), they were dehydrated by 10% increments of EtOH until reaching 100%. The samples were then critical point dried, sputter coated for 2 min with 60:40 AuPd and visualized with a ThermoApreo APREO VS SEM at 2 kV and 25pA in Immersion mode. Samples for TEM were gradually dehydrated to 100%EtOH, embedded in Spurr’s, sectioned to silver-gold thickness, and visualized at 80 keV on a Philips 410 TEM equipped with a 1 k x 1 k Advantage HR CCD camera from Advanced Microscopy Techniques (AMT).

## Supplementary Information

Supplementary Figures.

Supplementary Video S1.

Supplementary Video S2.

Supplementary Legends.
