# Supplementary material for: Sperm lacking Bindin are infertile but are otherwise indistinguishable from wildtype sperm
Source: Sci Rep. 2021 Nov 3;11:21583. doi: 10.1038/s41598-021-00570-6 (PMC8566474; doi:10.1038/s41598-021-00570-6)
Supplement: Supplementary file 1 — Supplementary Figures. [file 41598_2021_570_MOESM1_ESM.pptx]

## Slide 1
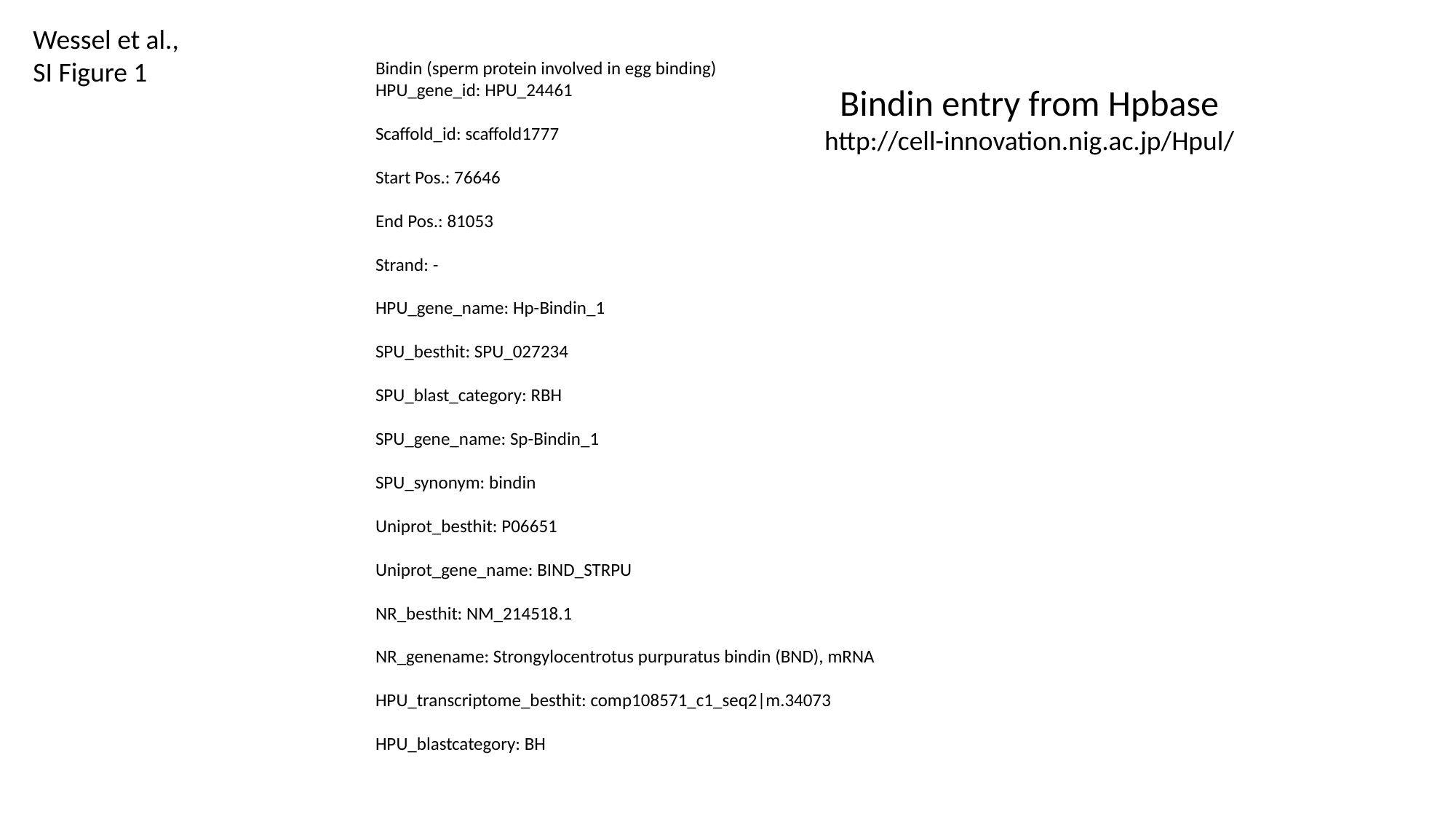

Wessel et al.,
SI Figure 1
Bindin (sperm protein involved in egg binding)
HPU_gene_id: HPU_24461
Scaffold_id: scaffold1777
Start Pos.: 76646
End Pos.: 81053
Strand: -
HPU_gene_name: Hp-Bindin_1
SPU_besthit: SPU_027234
SPU_blast_category: RBH
SPU_gene_name: Sp-Bindin_1
SPU_synonym: bindin
Uniprot_besthit: P06651
Uniprot_gene_name: BIND_STRPU
NR_besthit: NM_214518.1
NR_genename: Strongylocentrotus purpuratus bindin (BND), mRNA
HPU_transcriptome_besthit: comp108571_c1_seq2|m.34073
HPU_blastcategory: BH
Bindin entry from Hpbase
http://cell-innovation.nig.ac.jp/Hpul/

## Slide 2
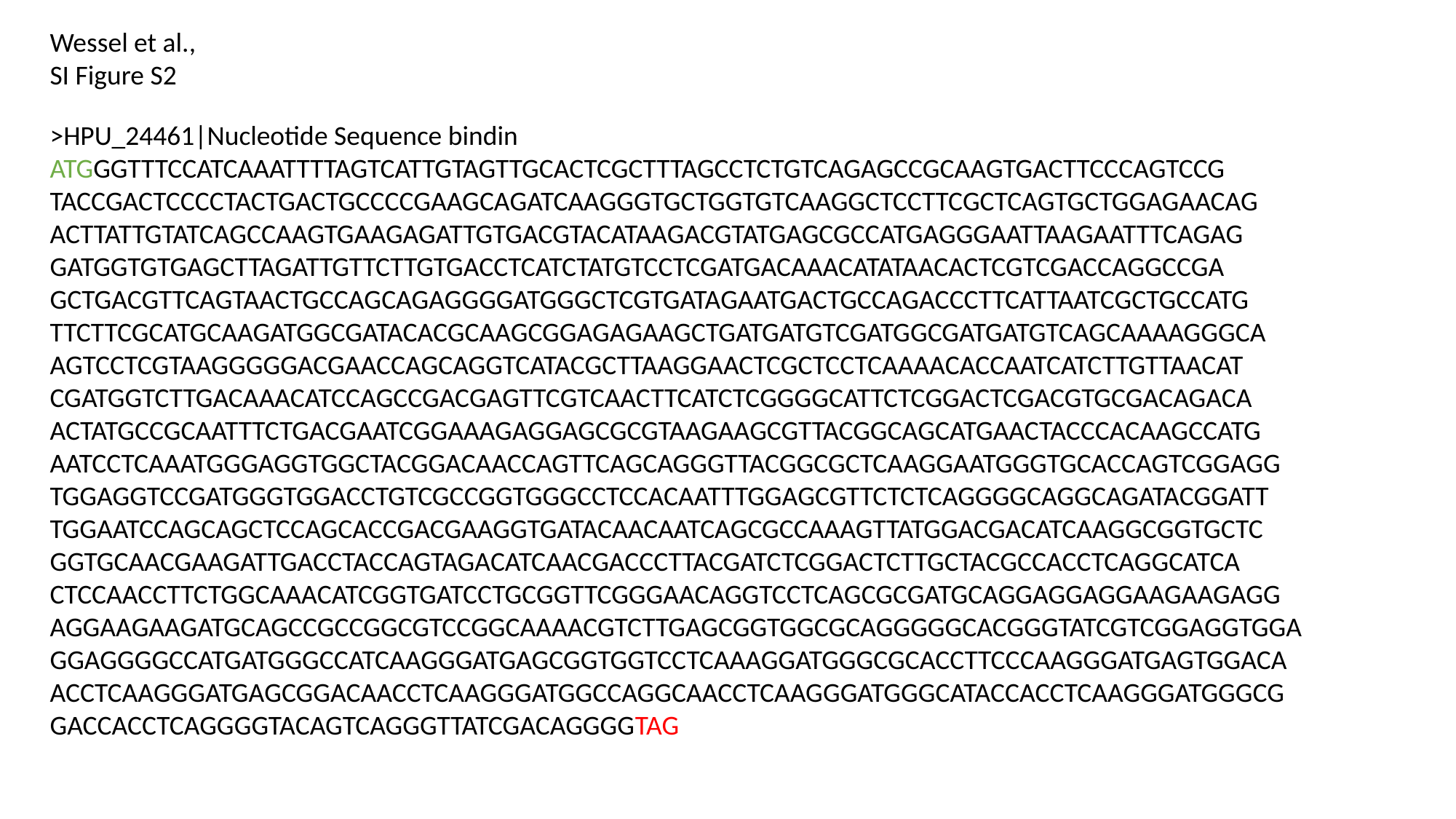

Wessel et al.,
SI Figure S2
>HPU_24461|Nucleotide Sequence bindin
ATGGGTTTCCATCAAATTTTAGTCATTGTAGTTGCACTCGCTTTAGCCTCTGTCAGAGCCGCAAGTGACTTCCCAGTCCG TACCGACTCCCCTACTGACTGCCCCGAAGCAGATCAAGGGTGCTGGTGTCAAGGCTCCTTCGCTCAGTGCTGGAGAACAG ACTTATTGTATCAGCCAAGTGAAGAGATTGTGACGTACATAAGACGTATGAGCGCCATGAGGGAATTAAGAATTTCAGAG GATGGTGTGAGCTTAGATTGTTCTTGTGACCTCATCTATGTCCTCGATGACAAACATATAACACTCGTCGACCAGGCCGA GCTGACGTTCAGTAACTGCCAGCAGAGGGGATGGGCTCGTGATAGAATGACTGCCAGACCCTTCATTAATCGCTGCCATG TTCTTCGCATGCAAGATGGCGATACACGCAAGCGGAGAGAAGCTGATGATGTCGATGGCGATGATGTCAGCAAAAGGGCA AGTCCTCGTAAGGGGGACGAACCAGCAGGTCATACGCTTAAGGAACTCGCTCCTCAAAACACCAATCATCTTGTTAACAT CGATGGTCTTGACAAACATCCAGCCGACGAGTTCGTCAACTTCATCTCGGGGCATTCTCGGACTCGACGTGCGACAGACA ACTATGCCGCAATTTCTGACGAATCGGAAAGAGGAGCGCGTAAGAAGCGTTACGGCAGCATGAACTACCCACAAGCCATG AATCCTCAAATGGGAGGTGGCTACGGACAACCAGTTCAGCAGGGTTACGGCGCTCAAGGAATGGGTGCACCAGTCGGAGG TGGAGGTCCGATGGGTGGACCTGTCGCCGGTGGGCCTCCACAATTTGGAGCGTTCTCTCAGGGGCAGGCAGATACGGATT TGGAATCCAGCAGCTCCAGCACCGACGAAGGTGATACAACAATCAGCGCCAAAGTTATGGACGACATCAAGGCGGTGCTC GGTGCAACGAAGATTGACCTACCAGTAGACATCAACGACCCTTACGATCTCGGACTCTTGCTACGCCACCTCAGGCATCA CTCCAACCTTCTGGCAAACATCGGTGATCCTGCGGTTCGGGAACAGGTCCTCAGCGCGATGCAGGAGGAGGAAGAAGAGG AGGAAGAAGATGCAGCCGCCGGCGTCCGGCAAAACGTCTTGAGCGGTGGCGCAGGGGGCACGGGTATCGTCGGAGGTGGA GGAGGGGCCATGATGGGCCATCAAGGGATGAGCGGTGGTCCTCAAAGGATGGGCGCACCTTCCCAAGGGATGAGTGGACA ACCTCAAGGGATGAGCGGACAACCTCAAGGGATGGCCAGGCAACCTCAAGGGATGGGCATACCACCTCAAGGGATGGGCG GACCACCTCAGGGGTACAGTCAGGGTTATCGACAGGGGTAG

## Slide 3
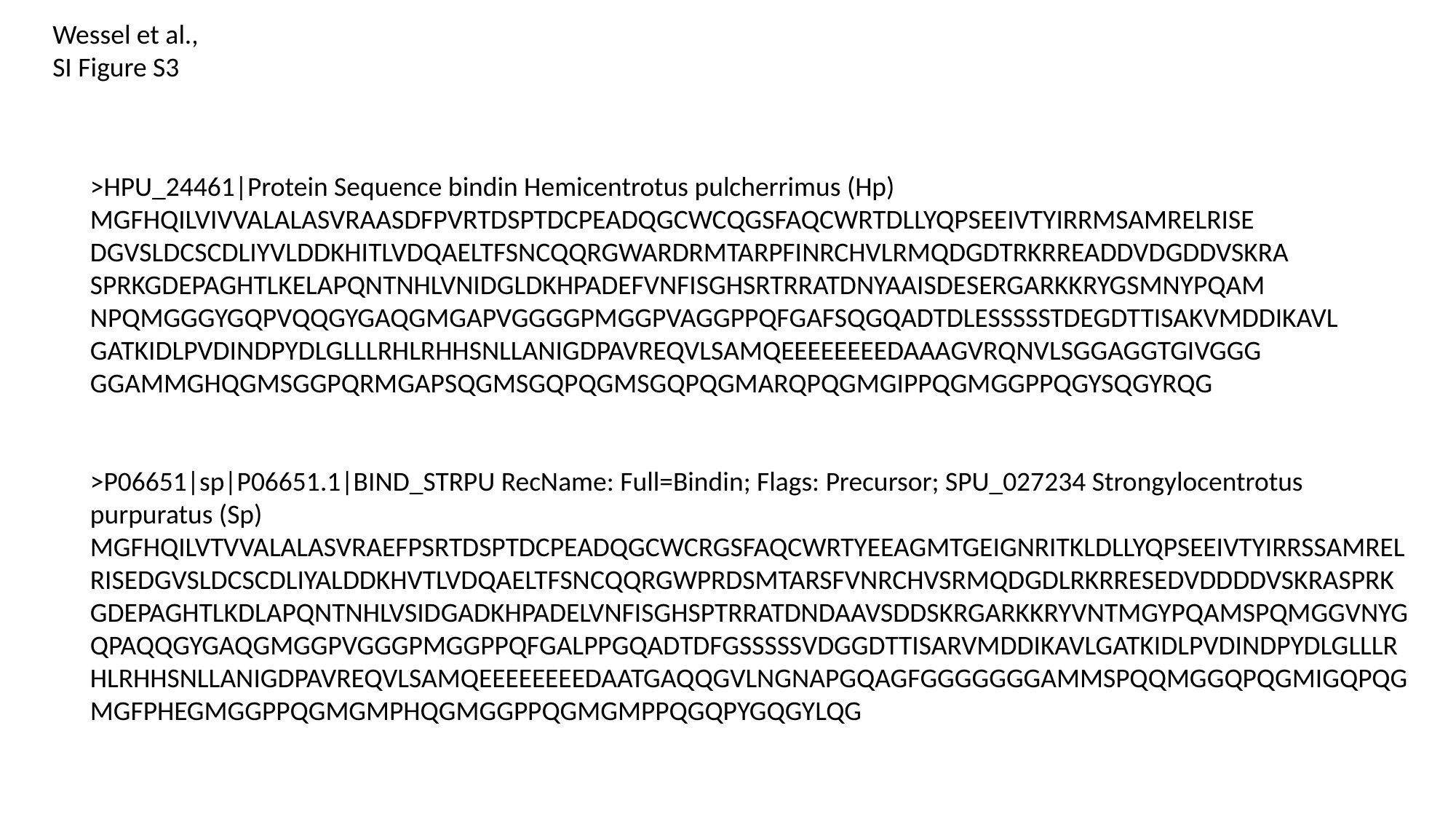

Wessel et al.,
SI Figure S3
>HPU_24461|Protein Sequence bindin Hemicentrotus pulcherrimus (Hp)
MGFHQILVIVVALALASVRAASDFPVRTDSPTDCPEADQGCWCQGSFAQCWRTDLLYQPSEEIVTYIRRMSAMRELRISE DGVSLDCSCDLIYVLDDKHITLVDQAELTFSNCQQRGWARDRMTARPFINRCHVLRMQDGDTRKRREADDVDGDDVSKRA SPRKGDEPAGHTLKELAPQNTNHLVNIDGLDKHPADEFVNFISGHSRTRRATDNYAAISDESERGARKKRYGSMNYPQAM NPQMGGGYGQPVQQGYGAQGMGAPVGGGGPMGGPVAGGPPQFGAFSQGQADTDLESSSSSTDEGDTTISAKVMDDIKAVL GATKIDLPVDINDPYDLGLLLRHLRHHSNLLANIGDPAVREQVLSAMQEEEEEEEEDAAAGVRQNVLSGGAGGTGIVGGG GGAMMGHQGMSGGPQRMGAPSQGMSGQPQGMSGQPQGMARQPQGMGIPPQGMGGPPQGYSQGYRQG
>P06651|sp|P06651.1|BIND_STRPU RecName: Full=Bindin; Flags: Precursor; SPU_027234 Strongylocentrotus purpuratus (Sp)
MGFHQILVTVVALALASVRAEFPSRTDSPTDCPEADQGCWCRGSFAQCWRTYEEAGMTGEIGNRITKLDLLYQPSEEIVTYIRRSSAMRELRISEDGVSLDCSCDLIYALDDKHVTLVDQAELTFSNCQQRGWPRDSMTARSFVNRCHVSRMQDGDLRKRRESEDVDDDDVSKRASPRKGDEPAGHTLKDLAPQNTNHLVSIDGADKHPADELVNFISGHSPTRRATDNDAAVSDDSKRGARKKRYVNTMGYPQAMSPQMGGVNYGQPAQQGYGAQGMGGPVGGGPMGGPPQFGALPPGQADTDFGSSSSSVDGGDTTISARVMDDIKAVLGATKIDLPVDINDPYDLGLLLRHLRHHSNLLANIGDPAVREQVLSAMQEEEEEEEEDAATGAQQGVLNGNAPGQAGFGGGGGGGAMMSPQQMGGQPQGMIGQPQGMGFPHEGMGGPPQGMGMPHQGMGGPPQGMGMPPQGQPYGQGYLQG

## Slide 4
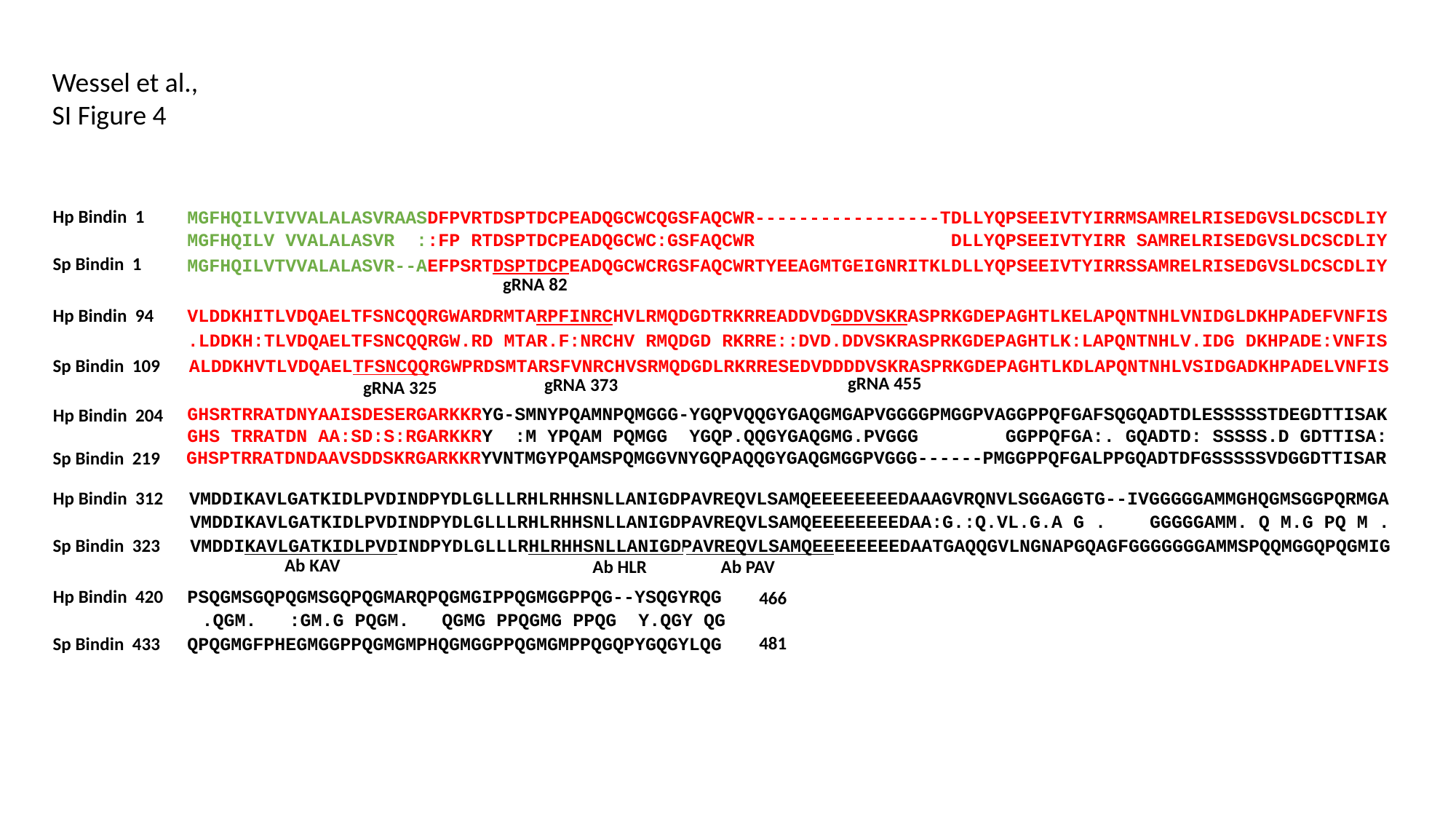

Wessel et al., SI Figure 4
Hp Bindin 1
MGFHQILVIVVALALASVRAASDFPVRTDSPTDCPEADQGCWCQGSFAQCWR-----------------TDLLYQPSEEIVTYIRRMSAMRELRISEDGVSLDCSCDLIY
MGFHQILV VVALALASVR ::FP RTDSPTDCPEADQGCWC:GSFAQCWR DLLYQPSEEIVTYIRR SAMRELRISEDGVSLDCSCDLIY
Sp Bindin 1
MGFHQILVTVVALALASVR--AEFPSRTDSPTDCPEADQGCWCRGSFAQCWRTYEEAGMTGEIGNRITKLDLLYQPSEEIVTYIRRSSAMRELRISEDGVSLDCSCDLIY
gRNA 82
VLDDKHITLVDQAELTFSNCQQRGWARDRMTARPFINRCHVLRMQDGDTRKRREADDVDGDDVSKRASPRKGDEPAGHTLKELAPQNTNHLVNIDGLDKHPADEFVNFIS
Hp Bindin 94
.LDDKH:TLVDQAELTFSNCQQRGW.RD MTAR.F:NRCHV RMQDGD RKRRE::DVD.DDVSKRASPRKGDEPAGHTLK:LAPQNTNHLV.IDG DKHPADE:VNFIS
ALDDKHVTLVDQAELTFSNCQQRGWPRDSMTARSFVNRCHVSRMQDGDLRKRRESEDVDDDDVSKRASPRKGDEPAGHTLKDLAPQNTNHLVSIDGADKHPADELVNFIS
Sp Bindin 109
gRNA 455
gRNA 373
gRNA 325
GHSRTRRATDNYAAISDESERGARKKRYG-SMNYPQAMNPQMGGG-YGQPVQQGYGAQGMGAPVGGGGPMGGPVAGGPPQFGAFSQGQADTDLESSSSSTDEGDTTISAK
Hp Bindin 204
GHS TRRATDN AA:SD:S:RGARKKRY :M YPQAM PQMGG YGQP.QQGYGAQGMG.PVGGG GGPPQFGA:. GQADTD: SSSSS.D GDTTISA:
GHSPTRRATDNDAAVSDDSKRGARKKRYVNTMGYPQAMSPQMGGVNYGQPAQQGYGAQGMGGPVGGG------PMGGPPQFGALPPGQADTDFGSSSSSVDGGDTTISAR
Sp Bindin 219
Hp Bindin 312
VMDDIKAVLGATKIDLPVDINDPYDLGLLLRHLRHHSNLLANIGDPAVREQVLSAMQEEEEEEEEDAAAGVRQNVLSGGAGGTG--IVGGGGGAMMGHQGMSGGPQRMGA
VMDDIKAVLGATKIDLPVDINDPYDLGLLLRHLRHHSNLLANIGDPAVREQVLSAMQEEEEEEEEDAA:G.:Q.VL.G.A G . GGGGGAMM. Q M.G PQ M .
VMDDIKAVLGATKIDLPVDINDPYDLGLLLRHLRHHSNLLANIGDPAVREQVLSAMQEEEEEEEEDAATGAQQGVLNGNAPGQAGFGGGGGGGAMMSPQQMGGQPQGMIG
Sp Bindin 323
Ab KAV
Ab HLR
Ab PAV
Hp Bindin 420
PSQGMSGQPQGMSGQPQGMARQPQGMGIPPQGMGGPPQG--YSQGYRQG
466
.QGM. :GM.G PQGM. QGMG PPQGMG PPQG Y.QGY QG
481
Sp Bindin 433
QPQGMGFPHEGMGGPPQGMGMPHQGMGGPPQGMGMPPQGQPYGQGYLQG

## Slide 5
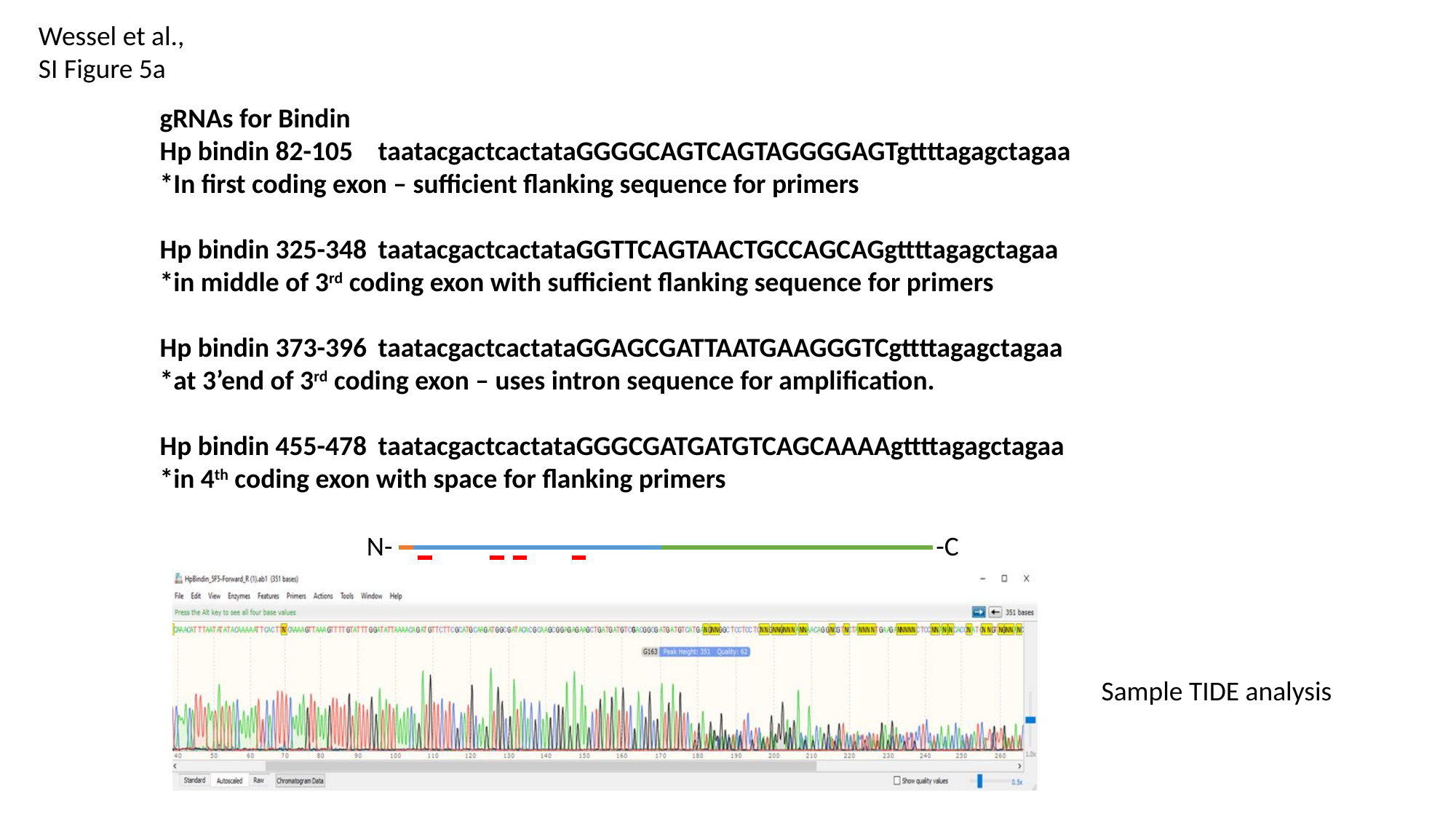

Wessel et al.,
SI Figure 5a
gRNAs for Bindin
Hp bindin 82-105 	taatacgactcactataGGGGCAGTCAGTAGGGGAGTgttttagagctagaa
*In first coding exon – sufficient flanking sequence for primers
Hp bindin 325-348 	taatacgactcactataGGTTCAGTAACTGCCAGCAGgttttagagctagaa
*in middle of 3rd coding exon with sufficient flanking sequence for primers
Hp bindin 373-396 	taatacgactcactataGGAGCGATTAATGAAGGGTCgttttagagctagaa
*at 3’end of 3rd coding exon – uses intron sequence for amplification.
Hp bindin 455-478 	taatacgactcactataGGGCGATGATGTCAGCAAAAgttttagagctagaa
*in 4th coding exon with space for flanking primers
-C
N-
Sample TIDE analysis

## Slide 6
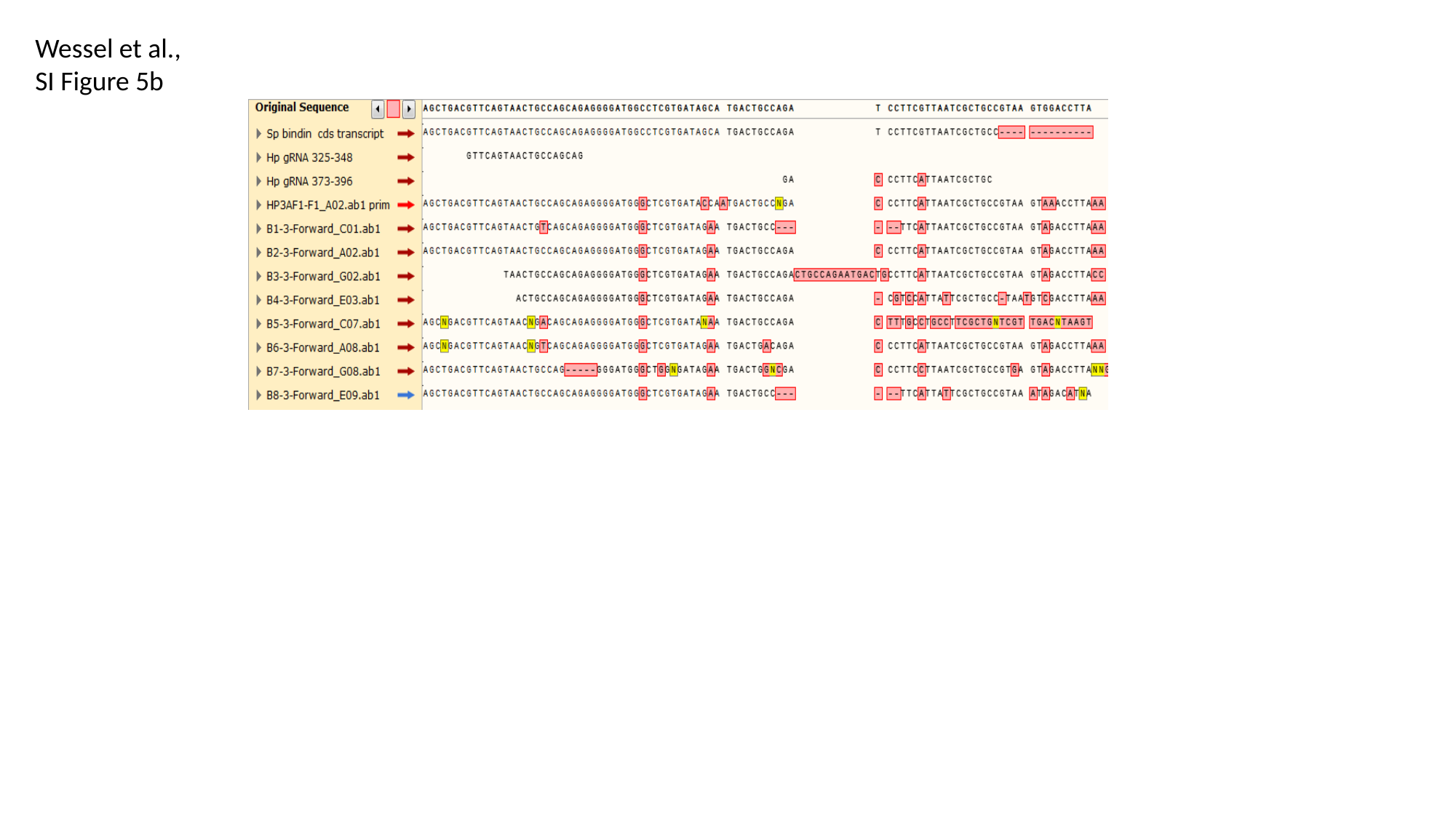

Wessel et al.,
SI Figure 5b

## Slide 7
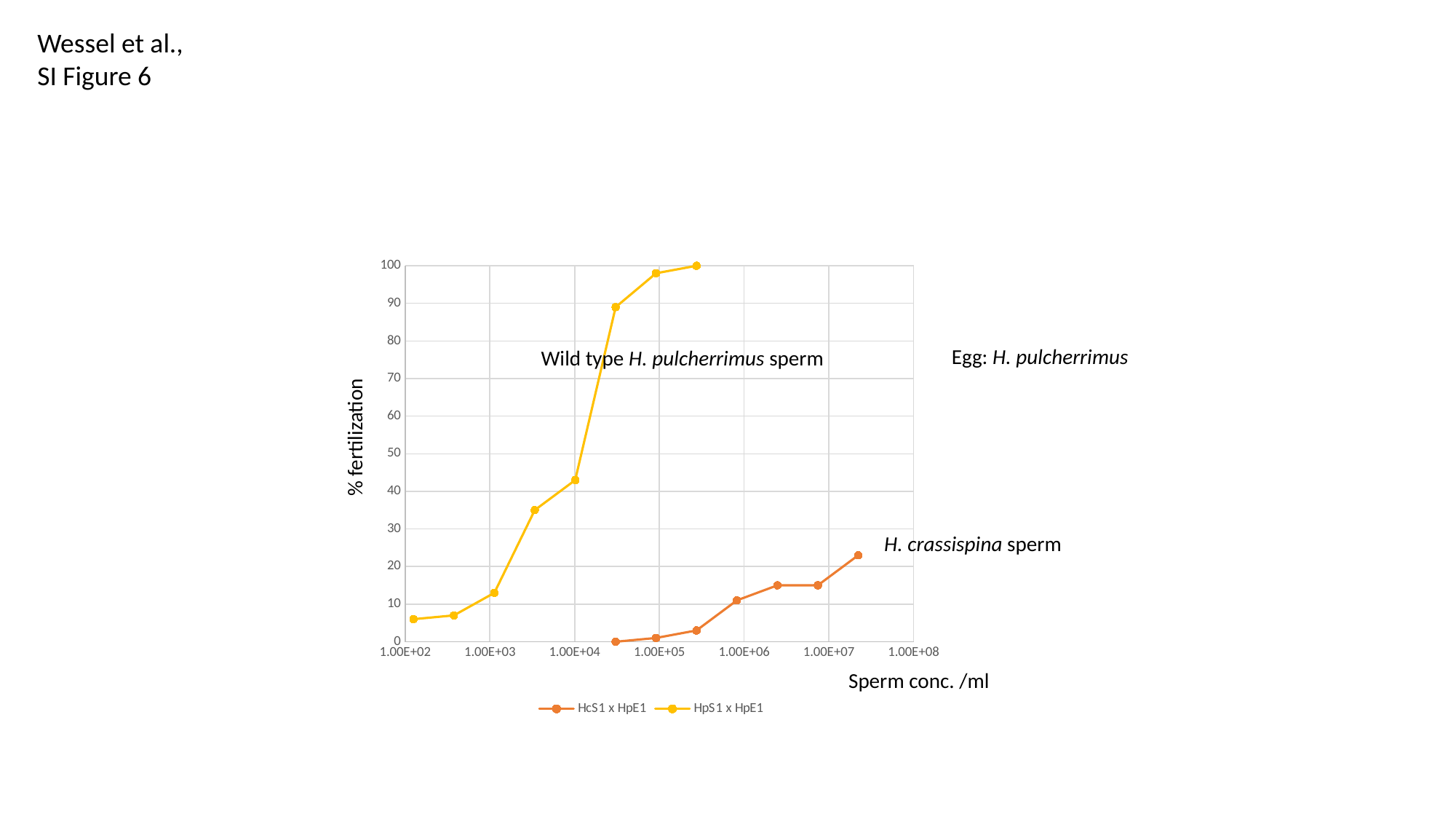

Wessel et al.,
SI Figure 6
### Chart
| Category | HcS1 x HpE1 | HpS1 x HpE1 |
|---|---|---|Egg: H. pulcherrimus
Wild type H. pulcherrimus sperm
% fertilization
H. crassispina sperm
Sperm conc. /ml

## Slide 8
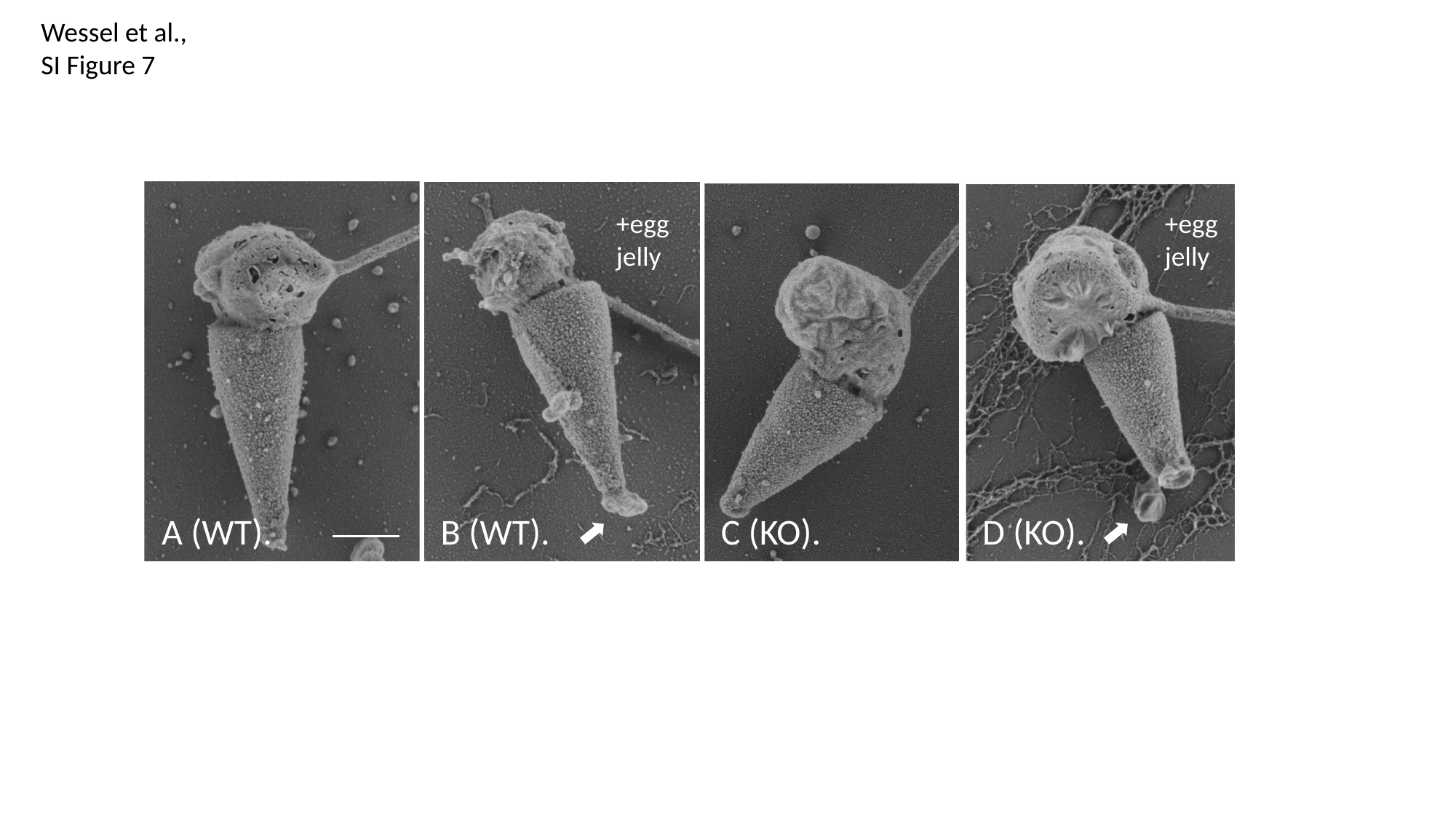

Wessel et al.,
SI Figure 7
+egg jelly
+egg jelly
A (WT).
B (WT).
C (KO).
D (KO).

## Slide 9
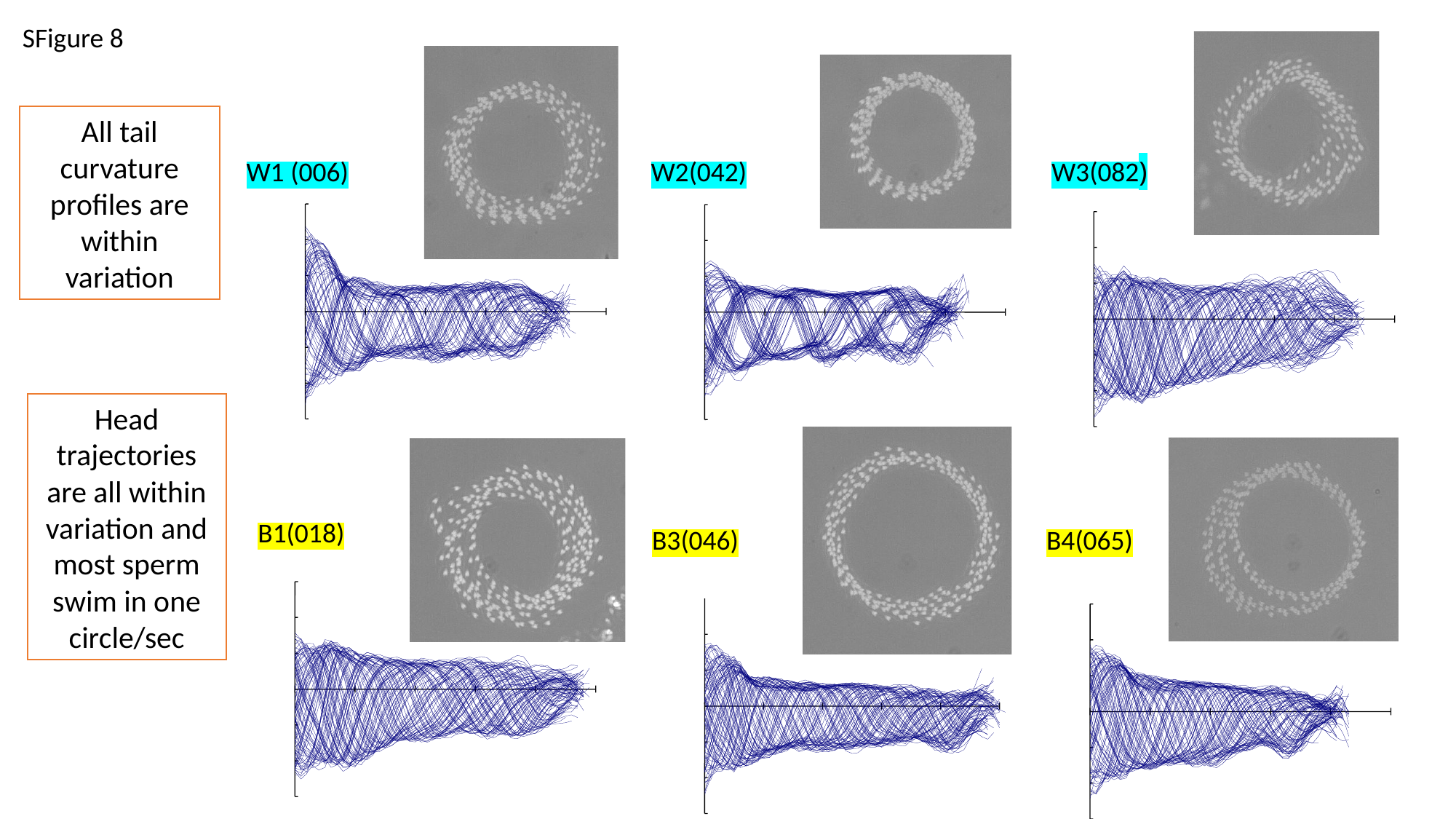

SFigure 8
All tail curvature profiles are within variation
W1 (006)
W2(042)
W3(082)
Head trajectories are all within variation and most sperm swim in one circle/sec
B1(018)
B3(046)
B4(065)

## Slide 10
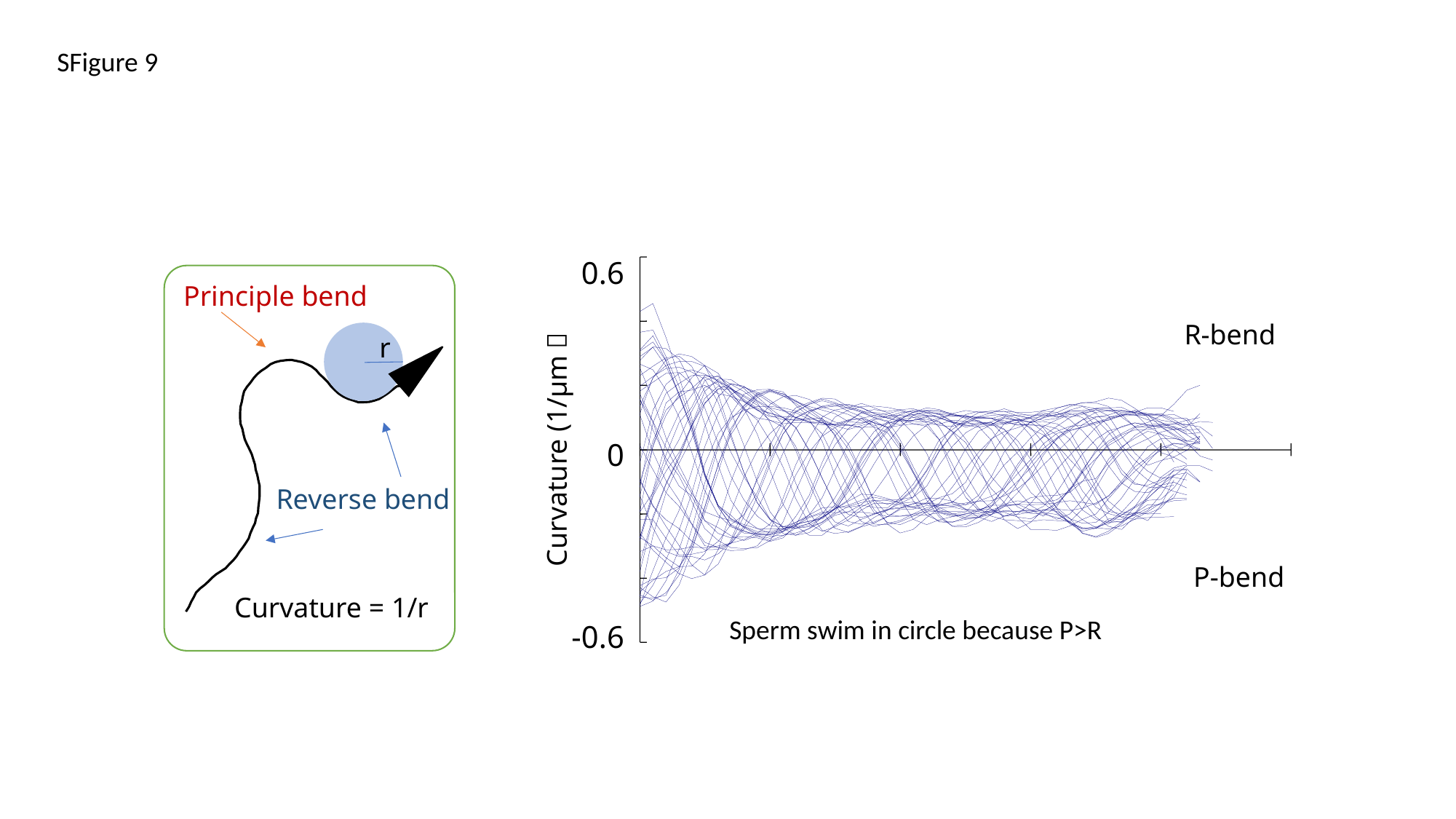

SFigure 9
0.6
0
-0.6
Principle bend
r
Reverse bend
Curvature = 1/r
R-bend
Curvature (1/µm）
P-bend
Sperm swim in circle because P>R

## Slide 11
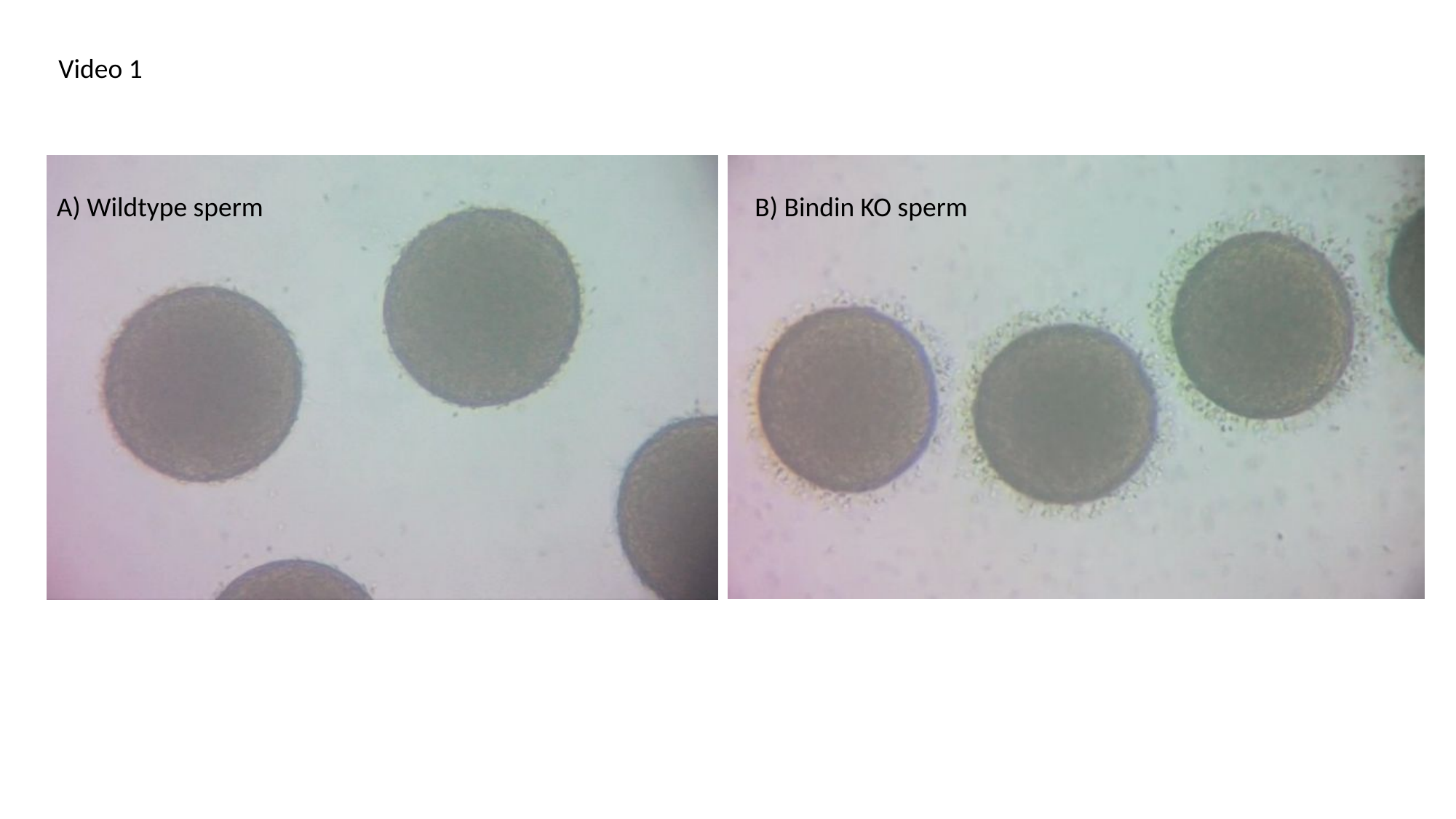

Video 1
B) Bindin KO sperm
A) Wildtype sperm

## Slide 12
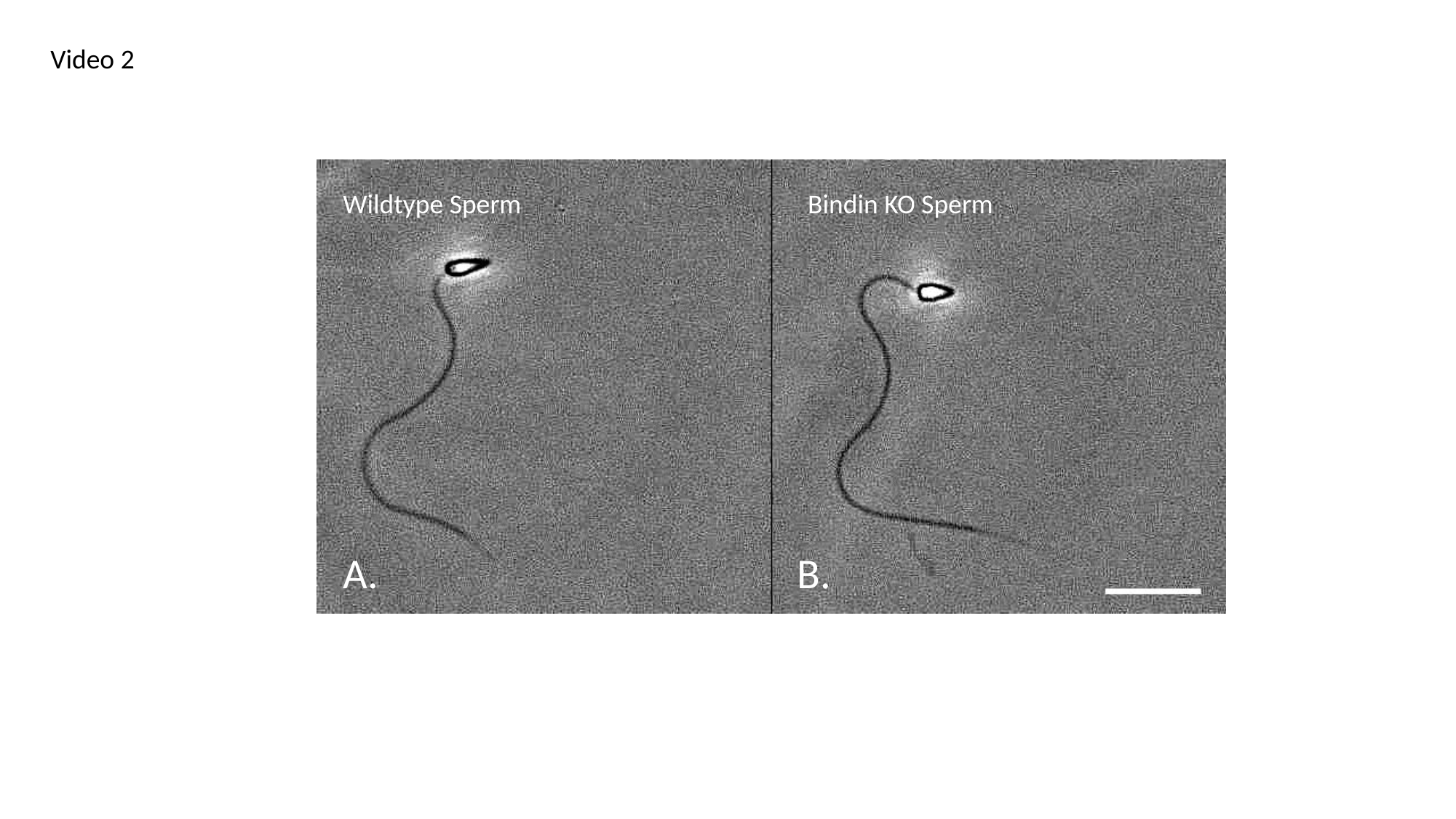

Video 2
Wildtype Sperm
Bindin KO Sperm
A.
B.
