# Supplementary material for: Sperm lacking Bindin are infertile but are otherwise indistinguishable from wildtype sperm
Source: Sci Rep. 2021 Nov 3;11:21583. doi: 10.1038/s41598-021-00570-6 (PMC8566474; doi:10.1038/s41598-021-00570-6)
Supplement: Supplementary file 4 — Supplementary Legends. [file 41598_2021_570_MOESM4_ESM.docx]

**Supplemental Figure Legends**

**SFigure 1:** Details of the *bindin* locus in HpBase [http://cell-innovation.nig.ac.jp/Hpul/]

**SFigure 2:** The nucleotide sequence of the *bindin* coding region. Predicted methionine codon in green and the stop codon in red.

**SFigure 3:** Predicted Bindin amino acid sequence derived from the genomes of two sea urchins *Hemicentrotus pulcherrimus* and *Strongylocentrotus purpuratus.*

**SFigure 4:** Sequence alignment of *Hemicentrotus pulcherrimus* (Hp; top line) and *Strongylocentrotus purpuratus* (Sp; bottom line) with identical amino acids indicated by the middle line. Signal sequence of pre-pro-protein is in green, the pro-protein domain is in red, and the mature Bindin region is shown in black. The sites in the genome targeted by the gRNAs are underlined (gRNA 82, gRNA 325, gRNA 373, and gRNA 455).

**SFigure 5:** **A)** Guide RNAs (gRNAs) used for mutagenesis as derived from the prediction algorithm CRISPRscan (CRISPRscan.org). Middle diagram giving relative placements of the gRNAs in the pro-protein domain of the full-length Bindin protein. Bottom diagram shows DNA sequencing of a PCR-amplicon derived from Bindin animal #5, with site of gRNA 373 (blue bar). Precipitous degradation of the sequencing chromatogram indicates gRNA-directed mutation. **B)** Snapgene view of DNA sequences associated with gRNA 325 and gRNA 373. The yellow boxes on the left indicate the rows of sequences of both the gRNAs, the Hp genomic sequence (Original Sequence), Sp genomic sequence at the same locus, the gRNAs, and the DNA from the different Bindin adults. Note especially the changes in sequence at the site of gRNA 373 that includes deletions, changes, and insertions

**SFigure 6:** Sperm from two species (*Hemicentrotus pulcherrimus* and *Heliocidaris crassispina*) challenging eggs of *Hemicentrotus pulcherrimus*. Sperm from a different species of sea urchin is able to activate eggs of a different species, significantly more than the same species of sperm lacking Bindin (see Figure 2). This suggests that Bindin is the sole egg binding factor in these sea urchins.

**SFigure 7:** SEM of sperm in presence and absence of egg jelly. A and C are sperm in seawater from wildtype and *bindin* -/- sperm, respectively. B and D show fibrous strands resulting from egg jelly and sperm that have undergone the acrosome reaction (white arrows). On average, the *bindin-KO* spermhead is 8% shorter than the wildtype head, as measured in SEM (n= 12). Bar = 1 um.

**SFigure 8:** All tail curvature profiles and head trajectories are within normal variation of each other.

**SFigure 9:** Methodology of tail curvature metrics.

**Video Legends**

**Supplemental Video 1:** Hp-*bindin* -/- sperm do not bind to nor activate eggs. A and B, Wildtype eggs challenged with *bindin* -/- sperm after 10 minutes incubation or wildtype sperm immediately after the sperm addition (membrane rising 30 seconds after sperm addition) shows robust fertilization envelope with wildtype sperm but not with *bindin* -/- sperm.

**Supplemental Video 2:** Swimming sperm of wildtype (left) and *bindin* KO (right) *Hemicentrotus pulcherrimus*. Image contrast has been enhanced using Image-J (ver.1.52a). Note that sperm from *bindin* KO sea urchin did not show any obvious defect in flagellar movement. Play speed: 10 times slow. Scale bar: 10 μm.
